# The influence of oral health conditions, socioeconomic status and home environment factors on schoolchildren's self-perception of quality of life

**DOI:** 10.1186/1477-7525-10-6

**Published:** 2012-01-13

**Authors:** Janice S Paula, Isabel CG Leite, Anderso B Almeida, Glaucia MB Ambrosano, Antônio C Pereira, Fábio L Mialhe

**Affiliations:** 1 Department of Community Dentistry, Division of Health Education and Health Promotion, Piracicaba Dental School, P.O. BOX 52, University of Campinas -UNICAMP, 13414-903, Piracicaba, SP, Brazil; 2 Department of Public Health, Medicine School, Federal University of Juiz de Fora (UFJF) Juiz de Fora, MG, Brazil

## Abstract

**Background:**

The objective this study was to investigate the influence of clinical conditions, socioeconomic status, home environment, subjective perceptions of parents and schoolchildren about general and oral health on schoolchildren's oral health-related quality of life (OHRQoL).

**Methods:**

A sample of 515 schoolchildren, aged 12 years was randomly selected by conglomerate analysis from public and private schools in the city of Juiz de Fora, Brazil. The schoolchildren were clinically examined for presence of caries lesions (DMFT and dmft index), dental trauma, enamel defects, periodontal status (presence/absence of bleeding), dental treatment and orthodontic treatment needs (DAI). The SiC index was calculated. The participants were asked to complete the Brazilian version of Child Perceptions Questionnaire (CPQ_11-14_) and a questionnaire about home environment. Questions were asked about the presence of general diseases and children's self-perception of their general and oral health status. In addition, a questionnaire was sent to their parents inquiring about their socioeconomic status (family income, parents' education level, home ownership) and perceptions about the general and oral health of their school-aged children. The chi-square test was used for comparisons between proportions. Poisson's regression was used for multivariate analysis with adjustment for variances.

**Results:**

Univariate analysis revealed that school type, monthly family income, mother's education, family structure, number of siblings, use of cigarettes, alcohol and drugs in the family, parents' perception of oral health of schoolchildren, schoolchildren's self perception their general and oral health, orthodontic treatment needs were significantly associated with poor OHRQoL (p < 0.001). After adjusting for potential confounders, variables were included in a Multivariate Poisson regression. It was found that the variables children's self perception of their oral health status, monthly family income, gender, orthodontic treatment need, mother's education, number of siblings, and household overcrowding showed a strong negative effect on oral health-related quality of life.

**Conclusions:**

It was concluded that the clinical, socioeconomic and home environment factors evaluated exerted a negative impact on the oral health-related quality of life of schoolchildren, demonstrating the importance of health managers addressing all these factors when planning oral health promotion interventions for this population.

## Background

Nowadays, researches point out the need to consider the functional and psychosocial dimensions of oral health for the implementation and evaluation of public health dentistry interventions. In order to achieve these dimensions, instruments that evaluate the oral health-related impact on quality of life (OHRQoL) have been developed [1,2], among them, the Child Perception Questionnaire (CPQ_11-14_) to assess OHRQoL at a specific age [3].

Several studies focused on children and adolescents have confirmed that oral diseases could have an impact on their quality of life [2,4-9], as caries lesions [10-14] and malocclusion [15-18].

However, a direct relationship between OHRQoL and clinical indicators should be interpreted with caution, because these impacts could be mediated by other factors, such personal, social, and environmental variables [2,19-21].

For example, the socioeconomic status of the household in which the children live may confound the relationships between oral health and OHRQoL [14,22,23]. This could occur because several studies have shown associations between low income and poor oral health [8,24-30].

Relative to the home environment, some studies have verified the influence of family on the oral health outcomes of children, considering that their families play a central role in promoting their oral health [31,32]. The parental perceptions of their children's oral health conditions may interfere in children's oral health [33]. Other studies have found that parents' socioeconomic characteristics are associated with their subjective perceptions related to their children's oral health status [33,34]. Therefore, the family environment may have an impact on children's self-perception about their OHRQoL, but there is scarcely any information on such association in the literature [14,32].

Although socioeconomic status and family environment could be linked to OHRQoL, this aspect has not yet been sufficiently investigated in studies to evaluate this association in schoolchildren. Only the research developed by Locker et al [22] studied the association between socioeconomic status and family structure on OHRQoL of schoolchildren. The authors verified that children with parents earning a low income, and with only one adult in the household had negative impact in their OHRQoL. In Brazil, only one study [23] evaluated the impact of socioeconomic factors, especially mothers' education, on OHRQoL.

In spite of these evidences, the hypothesis of the present study was that there were many other clinical, socioeconomic and home environment factors that could influence the OHRQoL of children, which have not yet been studied in a statistical regression model.

### Purpose

The objective this study was to investigate the influence of clinical conditions, socioeconomic status, home environment of children and subjective perceptions of parents and children about general and oral health on OHRQoL of schoolchildren.

## Methods

### Ethical issues

Prior to implementation, the research project was submitted to the Ethics Committee of the Piracicaba Dental School, University of Campinas, Brazil, and approved under Protocol 055/2009. Written informed consent was obtained from the participants or parents/guardians of the participants of this study.

### Study population

The present cross-sectional study referred to a representative sample of children from of Juiz de Fora, Brazil. Juiz de Fora is one of the pioneering cities in the industrial state of Minas Gerais, Brazil, and its predominating economic sectors are industry and services. The city has about 570,000 inhabitants, spread over a wide range of socio-economic backgrounds, of whom 98.91% have access to fluoridated water [35].

A total of 515 schoolchildren, 12 years of age, were examined according to a random multistage sampling design, which was considered representative of the city. The total number of schoolchildren at the age of 12 years was 7993 [35]. To calculate the probability sample, we adopted a 95% confidence interval level, 20% accuracy and design effect (deff) of 2. The sample size calculation was based on the DMFT (2.3) and standard deviation (2.72) of epidemiological survey previously conducted [36]. The schoolchildren were enrolled in public and private elementary schools and were included in a conglomerate analysis of a population-based study.

### Clinical examination

The schoolchildren were clinically examined at school by two calibrated examiners, in an outdoor setting, under natural light with ball-point probes and mirrors, according to the recommendations of the World Health Organization (WHO) for epidemiological surveys [37]. The examiner calibration process followed the WHO criteria and 20 children were examined in this phase. With regard to the questionnaire, as it has been validated, it was not necessary to conduct a pilot phase to implement them. The examiners were calibrated, and good intra-examiner reproducibility (Kappa > 0.91) was reached.

One examiner collected data with reference to the presence of decayed, missing, and filled teeth in the permanent and primary dentition (DMFT and dmft index). For statistical analysis, the presence or absence of untreated caries was evaluated according to the D component of DMFT index. Dental trauma, enamel defects (DDE index), periodontal status (bleeding) and dental treatment needs were evaluated in exams and categorized according to presence or absence, according WHO recommendations [37].

We used the WHO categorization of treatment needs and subsequently the data were dichotomized: zero, without treatment needs corresponding classification zero of the WHO criteria; and one, with treatment needs classification 1-9 of the WHO criteria [37].

The Significant Caries Index (SiC) was used to measure polarization of the occurrence of caries among participants of the tercile with higher DMF-T. The index was calculated according recommendations of Nishi et al [38].

The other examiner collected data on Malocclusion according the Dental Aesthetic Index (DAI), which assesses the dental appearance by collecting and attributing weight to 10 occlusal traits. The DAI score ranges from 13 (the most socially acceptable) to 100 (the least acceptable), and orthodontic treatment needs can be prioritized based on the predefined categories: having more acceptable dental appearance (score DAI < 31 - no orthodontic treatment need) or having less acceptable dental appearance (score DAI ≥ 31 - orthodontic treatment need) [39].

### Questionnaires

Data on the children's gender and the type of school at which they studied were collected. The participants were asked to complete two questionnaires. First, the Brazilian version of the Child Perceptions Questionnaire (CPQ_11-14_), developed by Jokovic et al [3]. This questionnaire was translated and validated for the Brazilian population by Barbosa et al [19] and presents good psychometric properties.

The CPQ _11-14 _is a self-administered instrument used to determine quality of life associated with oral health and consists of 35 items. The responses in each item are given using a Likert-type scale based on the number of points in the scale: "Never" = 0; "Once or twice" = 1; "Sometimes" = 2; "Often"= 3; and "Very often" = 4. Higher scores signify worse OHRQoL.

Secondly, a questionnaire was applied, asking questions about the presence of general diseases, the schoolchildren's self-perception of their general and oral health (excellent/very good/good or fair/poor) and home environment. The variables about home environment were: family structure (children live with both biological parents - yes/no), number of siblings (< 2 and 2 or more), use of cigarettes, alcohol and drugs in the family, and household overcrowding: number of people living in the household for number of rooms (≤ 1 person for room or > 1 person for room) [24].

In addition, a questionnaire was sent to their parents, asking questions about socioeconomic status (monthly family income, parents' educational level, home ownership - yes/no) [40] and their perception about their children's general and oral health. The monthly family income was measured on the basis of the number of minimum wages the family receives (up to 3/4 or more), considering the Brazilian minimum wage at time of data collection of approximately US $ 290 per month. The parents' educational level was categorized by number of years of schooling into two levels: up to 8 years of schooling or over 8 years.

### Statistical analyses

Data were analyzed using descriptive statistics, univariate analyses, and a regression model. The total score of the CPQ _11-14 _was dichotomized by the median, and represented the dependent variable being analyzed. The chi-square test was used for comparisons between proportions, and evaluated overall associations between the dependent and explanatory variables categorical. Poisson regression was used for multivariate analysis with adjustment for variances (significance of 5%). The statistical tests were performed using the SAS software [41].

A Poisson regression model was used to assess the association between the predictor variables and outcomes. A backward stepwise procedure was used to include or exclude explanatory variables in the adjustments for the models. Explanatory variables presenting a p value ≤ 0.20 in the assessment of association to each outcome (univariate analyses) were included in the adjustments for the model. Variables that were not related and did not contribute significantly to the model were eliminated and the final model contained only factors that remained associated at the level p ≤ 0.05.

## Results

According to conglomerate sampling, 363 (70.5%) students from public school and 152 (29.5%) from private schools participated in the survey. Examinations were carried out in 290 (56.3%) girls and in 225 (43.7%) boys. Of the examined participants, caries occurrence was observed in 85 subjects (16.5%); the mean DMFT was 1.09 (SD 1.70) and dmft was 0.85 (SD 1.42). The SiC index was 3.12.

The prevalence of bleeding was observed in 66 (12.82%) children and dental trauma in 17 (3.3%). Enamel defects were present in 81 (15.73%) participants. DAI scores ranged from 14.98 to 56.46 with a mean of 26.04 (SD 6.48), and 125 (24.3%) children presented orthodontic treatment needs (DAI ≥ 31).

The mean CPQ _11-14 _was 23.24 (SD 21.94) and median was 16, ranging from 0 to 106. Only 17 (3.3%) schoolchildren felt no impact on OHRQoL, with CPQ_11-14 _scores of zero. As regards the children's self-perceptions, 459 (89.1%) considered their general health excellent, very good or good, and 349 (67.8%) evaluated oral heath as excellent, very good or good. Two hundred and two participants (42.7%) had some general diseases.

With regard to home environment, 322 (62.5%) schoolchildren lived with both biological parents, and 442 (85.8%) had two or more siblings. The use of cigarettes, alcohol and drugs in the family was related by 229 (44.5%) participants. The calculation of household overcrowding resulted in 439 (85.2%) of family living in a house with one or fewer persons per room.

Among parents, 286 completed the questionnaire. As regards socioeconomic status, 242 (84.61%) related 3 or less minimum wages as their monthly family income and 156 (54.5%) reported home ownership. It was observed that 141 (49.3%) of children's mothers and 123 (43.35%) of their fathers had a higher educational level.

With regard to parents' perception, 266 (93%) considered their children's general health as excellent, very good or good, and 184 (65%) considered their children's oral health excellent, very good or good.

In TabbleTable 1 presents the socioeconomic and home environment variables that showed significant association with a score above the median in the CPQ_11-14_. In Table 2, associations were observed between clinical conditions and OHRQoL. There was a strong association between orthodontic treatment need and a score above the median in the CPQ_11-14 _(p < 0.001). The variables DMFT, decayed component (presence of cavitated caries lesion), dental treatment need and presence of bleeding also showed associations with worse OHRQoL (P < 0.05) in the schoolchildren.

**Table 1:** Univariate analysis of association between socioeconomic status and family environment variables with oral health- related quality of life in the overall median scores of CPQ_11-14 _(n = 515).

	**Variable**	**N**	**CPQ_11-14_**		**Prevalence ratio (PR)**		
			
			**Scores > median**		**PR crude**	**CI - 95%**	**p**
			**N**	**%**			
	
Gender	Female	290	155	53.4	1.21	1.01-1.46	0.0208
	Male	225	99	44.0	1.00		
School type	Public	363	225	62.0	3.64	2.60-5.08	< 0.0001
	Private	152	29	19.1	1.00		
Monthly family income	≤ 3 minimum wages	242	143	59.1	4.33	2.04-9.18	< 0.0001
	> 3 minimum wages	44	6	13.6	1.00		
Father's education	≤ 8 years	124	74	59.7	1.53	1.16-2.02	0.0012
	> 8 years	108	42	38.9	1.00		
Mother's education	≤ 8 years	141	94	66.7	1.82	1.42-2.33	< 0.0001
	> 8 years	142	52	36.6	1.00		
Children lives with both biological parents	No	193	116	60.1	1.40	1.18-1.66	0.0001
	Yes	322	138	42.9	1.00		
Number of siblings	2 or more	259	157	60.6	1.60	1.33-1.92	< 0.0001
	< 2	256	97	37.9	1.00		
Household overcrowding	More 1 person/room	76	50	65.8	1.42	1.17-1.71	0.0014
	≤ 1 person/room	439	204	46.5	1.00		
Cigarettes, alcohol and drug use	Yes	229	129	56.3	1.53	1.27-1.86	< 0.0001
	No	286	105	43.7	1.00		
Parents' perception of children's oral health	fair/poor	102	72	70.6	1.69	1.37-2.08	< 0.0001
	excellent/very good/good	184	77	41.8	1.00		
Children's perception of their general health	fair/poor	56	42	75.0	1.62	1.36-1.95	< 0.0001
	excellent/very good/good	459	212	46.2	1.00		
Children's perception of their oral health	fair/poor	166	124	74.7	2.01	1.70-2.36	< 0.0001
	excellent/very good/good	349	130	37.2	1.00		
General diseases	Yes	202	114	54.4	1.26	1.06-1.50	0.0061
	No	313	140	44.7	1.00		

**Table 2:** Univariate analysis of association between clinical condition variables and oral health- related quality of life in the overall median of CPQ_11-14 _(n = 515).

	**Variable**	**n**	**CPQ_11-14_**		**Prevalence ratio (PR)**		
			
			**scores > median**		**PR crude**	**CI - 95%**	**p**
			**N**	**%**			
	
DMFT	> 0	200	109	54.5	1.18	0.99-1.41	0.0373
	< 0	315	145	46.6	1.00		
D (caries lesion)	Present	85	49	57.6	1.21	0.98-1.49	0.0592
	absence	430	205	47.7	1.00		
Dental treatment need	Yes	87	57	65.5	1.42	1.18-1.71	0.0007
	No	428	197	46.0	1.00		
Bleeding	Yes	66	46	69.7	1.50	1.25-1.82	0.0003
	No	449	208	46.3	1.00		
Orthodontic treatment need	Yes	125	80	64.0	1.43	1.21-1.70	0.0001
	No	390	174	44.6	1.00		

The variables that showed no statistically significant difference (p > 0.05) were excluded from Tables 1 and 2: parents' perception of children's general health, home ownership, dmft and components, dental trauma, enamel defects and SiC.

All statistically significant variables were included in the Poisson regression model. After adjusting them, it was found that children's self perception of their oral health status (p < 0.0001); monthly family income (p = 0.0001); gender, orthodontic treatment need, mother's education (p ≤ 0.01); number of siblings, and household overcrowding (p ≤ 0.05) showed a strong negative effect on schoolchildren's oral health-related quality of life (Table 3).

**Table 3:** Associations among sociodemographic, familiar environment and clinical condition variables with oral health- related quality of life in the overall median score of CPQ_11-14_, through the Poisson model for multiple regression analysis.

	**Variable**	**n**	**CPQ_11-14_**		**Poisson regression**			
			
			**scores > median**		**Estimative (b)**	**SE**	**PR - adjusted**	**p**
			**N**	**%**				
	
Children's perception of their oral health	fair/poor	166	124	74.7	0.1696	0.0371	1.18	< 0.0001
	excellent/very good/good	349	130	37.2				
Monthly family income	≤ 3 minimum wages	242	143	59.1	0.2015	0.0527	1.22	0.0001
	> 3 minimum wages	44	6	13.6				
Gender	Female	290	155	53.4	0.1108	0.035	1.12	0.0015
	Male	225	99	44.0				
Orthodontic treatment need	Yes	125	80	64.0	0.1183	0.0382	1.12	0.0019
	No	390	174	44.6				
Mother's education	≤ 8 years	141	94	66.7	0.1011	0.0393	1.11	0.01
	> 8 years	142	52	36.6				
Number of siblings	2 or more	259	157	60.6	0.0813	0.0377	1.08	0.0312
	< 2	256	97	37.9				
Household overcrowding	More than 1 person/room	76	50	65.8	0.1056	0.0491	1.11	0.0315
	≤ 1 person/room	439	204	46.5				

## Discussion

Descriptive analysis of the clinical data indicated that the population evaluated in this study had good oral conditions and the average DMFT indicated a better profile when compared within the Brazilian context. Data from the National epidemiological survey realized in 2010 indicated that the DMFT mean for 12-year-old schoolchildren was 2.1 [42], and in the city of Juiz de Fora the DMFT mean was 1.09. The polarization of caries was observed because only 17% of the participants presented dental treatment needs. The SiC index (3.12) found was lower than it was in other studies in a 12-year-old population in Brazil [25,28,43,44].

With regard to malocclusion, it was observed that 24.3% of schoolchildren needed orthodontic treatment. In other studies conducted in Brazil and other countries, using the same malocclusion index, and samples of children of a similar age to those of the present study, prevalence of orthodontic treatment was higher [15-17,45-47]. In the same way as dental caries, malocclusion is a multifactorial disease and various determinants can contribute to its prevalence in different populations [48].

By means of this study it was possible to evaluate the impact of objective and subjective variables, conditions, and socio-environmental status on schoolchildren's OHRQoL. We found an interesting datum: in spite of the sample examined presenting good oral health conditions the majority of participants (96.7%) reported some impact on their OHRQoL; that is, CPQ_11-14 _scores differing from zero. This fact highlights the importance of further studies to investigate other factors that may influence the quality of life of children, which are not related to clinical conditions or dental indicators [4,9,19,21].

According to the univariate analysis, variables such as structure (children living with both biological parents, number of siblings) and family conditions (household overcrowding) have strong influence on schoolchildren's self-perceptions of their oral health. These important data have not yet been investigated in other studies and a hypothesis for this association may be attributed to the relations between home environment (family structure) oral health status and oral health behaviors in children [9,29,31].

Moreover, an association was found between parents' perception about their children's oral health conditions and the OHRQoL perceived by the children (p < 0.0001) in the univariate analysis. These results highlight the influence of family values related to oral health care on children's subjective perceptions about their OHRQoL. Although other studies have verified the influence of family in children's behavior and knowledge in oral health [14,31-34,49-51], this was the first study that indicated the influence of family on children's OHRQoL.

After controlling the confounding variables, the Poisson regression statistical analysis allowed the variables to be adjusted and controlled to define which of them generated the greatest impact on OHRQoL. It was observed that there was a statistically significant association between OHRQoL and the number of siblings and household overcrowding. Nevertheless, it is the first study demonstrating that the number of siblings and household overcrowding were associated with children's OHRQoL. In this respect the present study differs from the others in literature, which associated the number of siblings with tooth brushing [31,51], and household overcrowding with oral health conditions [24].

It was also observed that the monthly family income and mother's education had a strong impact on children's OHRQoL, which was corroborated by the similar results founded by Locker et al [22], in a study conducted in Canada, and Piovesan et al [23] in Brazil.

Children living in families with higher incomes generally present better oral hygiene behaviors, access to health care and preventive interventions, providing them with a better quality of life [27,33,52].

With regard to the clinical variables, in regression analysis, only malocclusion remained as an important oral health characteristic that had a negative impact on the quality of life. This result demonstrated the strong influence exerted by dental esthetic aspects on the schoolchildren's OHRQoL. The literature demonstrates that dentofacial esthetics play an important role in social interaction and psychological well-being [15,17,53-55].

The low prevalence of the other clinical conditions in the children assessed may have contributed to the result no statistically significant found among these clinical conditions and OHRQoL in the Poisson regression. However, the continuous surveillance of dental caries, periodontal status, dental trauma and enamel defects by public health managers is essential for providing a life course perspective involving care, and preventing future dental extractions [56].

Gerritsen et al [57] in a meta-analyses study, found that tooth loss had an impact on the OHRQoL of adults and older adult population. Therefore, public health interventions with the aim of impact on schoolchildren's oral health could present consequences later in life and subsequently, impact on OHRQoL.

With regard to psychological variables, it was found that children who presented a bad self-perception of their oral health showed significant associations with CPQ_11-14 _scores above the median. According to Barbosa et al [19], the children's self-perception about oral health is one global rating in CPQ_11-14_, and the association with the overall score of the instrument determined the validity of schoolchildren's responses.

As described, the orthodontic treatment need was the only clinical variable that presented association with OHRQoL outcomes, and its strength of association was less than that of a variety of other personal, social and environmental variables, suggesting that the former was mediated by the others. These results corroborated the importance of the social diagnosis for the planning of health promotion interventions in all social environments in which children live their lives, in order to promote supportive environments for them, in addition to personal skills to maximize the possibility of leading healthy lives and reducing inequalities [22,23,57].

Therefore, it is important to reconsider the current biomedical and restricted paradigm on OHRQOL and to begin to think about the validity of contemporary conceptual models of disease and its consequences, emphasizing the importance of personal, social, and environmental factors in mediating patient-centered quality of life outcomes [58,59].

The data of this research should be interpreted within the context of some limitations. The study had a cross-sectional design, which made it difficult to evaluate the indicators of risk for OHRQoL. The measures of behavior and self-esteem, which might influence the oral health conditions and subjective perception of the schoolchildren, were not included. Moreover, the evaluation of CPQ_11-14 _for health domains would be interesting to better define the impacts on quality of life reported by schoolchildren.

## Conclusion

It was concluded that the clinical, socioeconomic and home environment factors evaluated exerted a negative impact in the oral health-related quality of life of schoolchildren, demonstrating the importance of health managers addressing all these factors when planning oral health promotion interventions. We suggest that oral health promotion strategies should involve subjective, social and environmental aspects in planning, action and evaluation. In addition, new longitudinal studies should be conducted to determine causal relationships to OHQoL.

## Competing interests

The authors declare that they have no competing interests.

## Authors' contributions

JSP participated in the conception and design of the study, data interpretation, data acquisition, and drafting the manuscript. ICGL contributed to the conception and design of the study. ABA contributed to the data collection. GMBA participated in data analyses. ACP contributed to critical revision of manuscript. FLM participated in the conception and design of the study and critical revision of manuscript. All authors read and approved the final manuscript.
